# Production of hydrogen using plastic waste via Aspen Hysys simulation

**DOI:** 10.1038/s41598-024-55079-5

**Published:** 2024-02-28

**Authors:** Chua Qi Yi, Muhammad Na’im Bin Haji Bujang Haji Bojeng, Siti Khadijah Binti Haji Kamis, Nabisab Mujawar Mubarak, Rama Rao Karri, Hazwan Azri

**Affiliations:** 1 Petroleum and Chemical Engineering, Faculty of Engineering, Universiti Teknologi Brunei, Bandar Seri Begawan, BE1410 Brunei; 2 Department of Chemistry, School of Chemical Engineering and Physical Sciences, Lovely Professional University, Jalandhar, Punjab, India; 3 INTI International University, 71800 Nilai, Negeri Sembilan Malaysia

**Keywords:** Hydrogen, Pyrolysis, Plastic waste, Aspen Hysys, Environmental sciences, Energy science and technology

## Abstract

Plastic waste is being manufactured for the production of hydrogen. The amount of plastic waste collected annually is 189,953 tonnes from adjacent nations like Indonesia and Malaysia. Polyethylene (PE), Polypropylene (PP), Polyethylene Terephthalate (PET), Polyvinyl chloride (PVC), and Polystyrene (PS) are the five most prevalent forms of plastic found in most waste. Pyrolysis, water gas shift and steam reforming reaction, and pressure swing adsorption are the three main phases utilized and studied. In this research, authors examines the energy consumption on every stage. The plastic waste can be utilized to manufacture many hydrocarbons using the pyrolysis reaction. For this process, fast pyrolysis is being used at a temperature of 500 °C. A neutralization process is also needed due to the presence of Hydrochloric acid from the pyrolysis reaction, with the addition of sodium hydroxide. This is being carried to prevent any damage to the reactor during the process. Secondly, the steam reforming process continues after the water gas shift reaction has produced steam and carbon monoxide, followed by carbon dioxide and hydrogen formation. Lastly, pressure swing adsorption is designed to extract H_2_S and CO_2_ from the water gas shift and steam reforming reaction for greater purity of hydrogen. From the simulation study, it is observed that using various types of plastic waste procured (total input of 20,000 kg per hour of plastics) from, Brunei Darussalam, Malaysia and Indonesia, can produce about 340,000 tons of Hydrogen per year. Additionally, the annual profit of the Hydrogen production is estimated to be between $ 271,158,100 and $ 358,480,200. As per the economic analysis, it can be said that its a good to start hydrogen production plant in these regions.

## Introduction

The discovery of plastics was an important achievement that increased the quality of life for individuals. Since their first usage in the early 1900s, plastics have replaced many different kinds of materials in the manufacture of consumer goods, including metals, woods and ceramics, due to their light weight, durability, corrosion resistance by most chemicals, variety of applications, simplicity of processing, and relatively inexpensive^1^. Plastics played a significant role in consumer society because of their great properties and diverse uses. Therefore, worldwide plastics manufacturing has continually increased in recent years, reaching 360 million tons in 2018^2–4^ Despite the benefits indicated above, environmental issues have arisen as a result of the buildup of plastic waste ever since the development of plastics. Plastic waste has become a major concern in every country as the population grows. Since the past few years, the amount of mixed plastic waste (MPW), which includes segregated auto-shredder waste (ASW), waste agricultural plastics (WAP), and plastic-containing municipal solid waste (MSW), has continuously increased, and the disposal of these plastic wastes has become a worldwide concern^3–5^. The traditional approach of landfilling is becoming more costly and undesirable in many communities. Since plastic waste does not completely degrade, it takes up landfill space for hundreds of years after being discarded. Waste incineration is becoming increasingly common; however, it is also typically expensive and causes difficulties with unwanted pollutants. A third option would be recycling, which involves converting waste into items that can be utilized^6^.

The ongoing impacts of plastics on the seas, rivers, and terrestrial habitats are increasing the environmental concern regarding waste plastics. Within this context, implementing efficient waste plastic management systems is a crucial that has received significant attention lately. They can be disposed of via landfills or incinerators. However, those methods may cause harm to the environment as carbon dioxide may be released. Plastic waste can fill a high volume of the waste stream since it has a low density, causing disposal difficulty. From 1950 to 2018, approximately 8.3 billion tonnes of products made from plastics were manufactured, whereas 6.3 billion tonnes of plastic garbage were collected. Unfortunately, only about 9% of the garbage was recycled, and the remaining 12% was burned with or without heat recovery. The remaining has been dumped into the environment by engineered landfills or covert dumping^7–9^. As a result of this issue, various ecologically friendly waste plastics management approaches have been developed. Even though plastic waste may be reused and recycled, it will eventually become trash^10,11^.

Primary recycling (in-plant recycling), secondary recycling (mechanical recycling), tertiary recycling (chemical recycling), and quaternary recycling (energy recovery) are the four basic types of recycling techniques. Mechanical recycling is frequently viewed as the best method for recovering plastics. This is undoubtedly true for goods such as blow-molded containers and pallet wrap film, which can be clearly distinguished by polymer type and removed from the waste stream. However, this is not applicable to the vast amount of mixed plastic waste. Mixed waste plastics include various materials with diverse qualities designed for each respective purpose. Low-density polyethylene (LDPE), high-density polyethylene (HDPE), polypropylene (PP), polystyrene (PS), polyvinyl chloride (PVC), and polyethylene terephthalate (PET) are the most common components of mixed plastic waste. As a result of the complexity of polymer materials and the increasing usage of flexible plastic films, detaching each plastic component for mechanical recycling has become increasingly difficult, expensive, and energy-consuming. Therefore, mechanical recycling does not offer the most environmentally friendly recovery method for mixed plastic garbage^12,13^. Chemical recycling is the only process, amongst four other techniques of recycling, that adhere to sustainable development principles since it results in the production of the raw materials from which plastics are initially manufactured. Chemical recycling is getting more recognition from scientists because it can recover the energy content of plastics as liquid and gas. Chemical recycling might be a solution to the energy issue. Ever since the industrial revolution, almost every activity on Earth has relied extensively on petroleum and natural gas sources, particularly power production, for industrial purposes.

Furthermore, fossil fuels remain the primary energy sources for transportation, agriculture, and housing. Unfortunately, petroleum is a non-renewable source that will be limited in the near future due to intensive usage for industrial operations as the only significant and affordable energy source. Significant initiatives have been made to generate inexpensive renewable energy to solve the issue of deteriorating fossil fuels. Pyrolysis, known as thermolysis, is among the most significant chemical recycling methods.

The transformation of plastic trash into other goods has gained much attention in attempting to increase the value of plastic waste. Several thermochemical techniques for valorizing waste plastics have been developed in recent years. The value of these valorization approaches derives from their ability to generate chemicals and fuels from many types of plastics and their compositions. Most hydrogen is obtained commercially using catalytic steam reforming of hydrocarbons such as natural gas, LPG, or naphtha to produce syngas. Following this, a water gas shift reaction may produce a mixture of hydrogen and carbon dioxide and further separation and purifying procedures. However, methods that generate hydrogen from different raw materials, particularly wastes and byproducts, are equally appealing due to the potential environmental and economic benefits^13,14^. Between many plastic waste valorization approaches, thermochemical technologies offer the highest prospects for implementation as they have been explored to pilot and demonstration scale. Researchers have discovered that plastic with long polymer chains may be fractured at high temperatures to create oligomers without oxygen. The term pyrolysis was commonly applied to this process, even though it generally refers to the thermochemical degradation of organic compounds at high temperatures without oxygen. Many recycling techniques are costly, energy-intensive, and result in low-quality goods. Pyrolysis is an environmentally friendly and sustainable waste management technology for treating polymeric solid waste comprising carbonaceous elements such as plastics and biomass. Pyrolysis is an intriguing method for recovering fuels and gases from plastic waste. It has therefore been extensively researched using a variety of reactor designs, including fluidized beds, batch reactors, spouted beds, screw kilns, and many more^15^.

This research investigates the performance, effectiveness, and reliability of a pyrolysis reaction to produce Hydrogen gas from plastic waste. Plastic pyrolysis produces condensable gases, solid char residue and hydrocarbon liquid. Initially, the pyrolysis reaction could be carried out at varying temperatures, reaction durations, and with or without catalysts. Pyrolysis of plastics can take place at low temperatures (400 °C), medium temperatures (400–600 °C), or high temperatures (> 600 °C). The pyrolysis reaction is conducted in a conical spouted bed reactor (CSBR), a novel gas–solid contact technology that works well for plastic flash pyrolysis. Thus, the intense circular motion of the particles, combined with the fast heat transfer rate of this reactor, reduces bed agglomeration difficulties produced by melted plastic. This study also explores the main benefits and problems of plastic waste pyrolysis. Since the amount of plastic waste is enormous and can be easily obtained, utilizing it as a feedstock is extremely encouraging in terms of waste utilization and lower cost.

## Methodology

### Raw materials

Plastic bags usage are completely banned in Brunei Darussalam, however plastic bottles and other plastic materials are extensively used. They end up as waste. Hence beside using plastic waste produced in Brunei Darussalam, Plastic waste can be collected from nearby countries, Malaysia and Indonesia for throw away prices as well as, due to low transport costs. The plastic waste that is collected mainly contains 5 different types of plastic. In addition, miscellaneous types of plastic are easier or harder to be recycled due to the different varieties of plastics.

#### Polyethylene

Polyethylene (PE) is a long ethylene chain with a molecular formula of CH_2_=CH_2_. In industries, monomers such as ethylene and propylene are known as olefins, where ethylene is considered the simplest olefin. Some methods to fabricate PE use organic peroxide as an initiator because it generates free radicals to polymerize ethylene. Other methods, such as transition metal catalysts, are also widely used in industry to fabricate PE with various properties using different mechanisms^16^.

Their density or their molecular weight usually classifies industrial PE. Density gradient columns and hydrostatic methods are ways to measure density and classify the different types of PE. The crystalline content is directly affected by the density contained and can be used to estimate the crystallinity in PE. Some common classification methods have been developed to identify different types of PE. Below is the classification of PE from the Society of Plastics Industry (SPI) based on their density^16^:

- Low density: 0.910–0.925 g/cm^3^.
- Medium density: 0.926–0.940 g/cm^3^.
- High density: 0.941–0.965 g/cm^3^.

Similar to PSI, the American Society for Testing and Materials (ASTM) also had classification for PE based on their density but with additional classification. Below is the classification from ASTM for different densities of PE^16^:

- High-density polyethylene (HDPE): > 0.941 g/cm^3^.
- Linear medium density polyethylene (LMDPE): 0.926–0.941 g/cm^3^.
- Medium density polyethylene (MDPE): 0.926–0.941 g/cm^3^.
- Linear low-density polyethylene (LLDPE): 0.919–0.925 g/cm^3^.
- Low-density polyethylene (LDPE): 0.910–0.925 g/cm^3^.

#### Polypropylene

Polypropylene (PP) is one of the world’s most famous plastics. The propylene monomer has a molecular formula of C_3_H_6,_ one of the simplest alkenes. There are 2 types of polypropylene; low molecular weight amorphous polypropylene, which has limited commercial uses and industrial polypropylene, which is more widely used. The former polypropylene is usually manufactured using free radical and cationic methods. In contrast, polypropylene is manufactured under specific conditions using a transition metal as the catalyst with a metal alkyl cocatalyst^17^.

While comparing polyethylene (PE) with PP, although PE costs less, PP has better properties, such as higher melting temperature (T_m_) and tensile strength. Like PE, PP is also thermoplastic with very good chemical resistance with strong rigidity. This means that PP can be melted and shaped into other shapes and sizes for recycling. Some fabrication ways to produce PP are through injection moulding, fibre extrusion and film extrusion. These methods account for almost all of the PP production^17^.

#### Polystyrene

Polystyrene (PS) is a transparent thermoplastic, solid plastic or rigid foam material. There are three major polystyrene types: polystyrene foam, regular polystyrene plastic and polystyrene film. Extruded Polystyrene (EPS) and expanded Polystyrene (XPS) are two forms of foam polystyrene. The most popular and widely used polystyrene products, such as packing peanuts and Styrofoam, are included in extruded polystyrene. On the other hand, expanded Styrofoam has a greater density and is frequently utilized in applications like creating architecture models. Co-polymers include some varieties of polystyrene material. It can be more impact-resistant when mixed with other materials, such as homopolymer polystyrene, which is frequently quite brittle. Vacuum forming of polystyrene film is another option for use in packaging. Films can be stretched into oriented polystyrene (OPS), which is more brittle than alternatives such as Polypropylene but less expensive.

There are various ways polystyrene can be produced and manufactured, firstly by using fractional distillation, where the distillation of hydrocarbon fuels with the addition of a catalyst is undergone and produces plastic. This process, for the polystyrene case, is known as polymerization. Secondly, polystyrene undergoes free radical polymerization of styrene (monomer of polystyrene). Using free-radical initiators, styrene is mixed with this and heated at 102 °C. A polymer that has undergone several steps of polymerization dissolves in the monomer or diluent solution. Under vacuum, the unreacted monomer and diluent flash off, producing high molecular weight polystyrene^18,19^.

#### Polyethylene terephthalate

Polyethylene terephthalate (PET) is a commonly known plastic used worldwide. However, in the natural environment, it is exceedingly tough to hydrolyse. PET plastic is a low-cost, portable, and sturdy material since it is easily moulded into an assortment of products used in a wide range of applications. It is considered the most versatile and trusted material. PET is made of two raw materials, the transesterification of ethylene glycol and terephthalic acid, under high temperature and low pressure, forming long polymer chains. The chains lengthen as the mixture gets denser. The reaction is terminated after the desired chain length has been reached. After fast cooling, the PET strands that resemble spaghetti are extruded into little PET pellets. It is then stretched when reheated to a molten stage, and slowly crystallizes once elevated, it starts becoming opaque, more rigid, and less flexible.

#### Polyvinyl chloride

Polyvinyl chloride (PVC) is one of the most ordinary plastics worldwide, after PET and PP. Naturally, it is white and very brittle plastic. There are two PVC types: rigid or unplasticized polymer and flexible plastic. PVC is distinguished by its brittle yet rigid structure at base form. PVC is mainly used as a rigid polymer since most industries use it for its application, such as plumbing, sewage, and agriculture^20^. Besides this, many PVC varieties are used; CPVC, PVC-O and PVC-M. CPVC, or chlorinated polyvinyl chloride or per chlorovinyl, is a PVC resin made using chlorination. Due to this, CPVC contains high chlorine, making it highly durable, chemically stable and retardant. It also has a wide range of temperatures. Secondly, molecular-oriented PVC or PVC-O is formed by converting the PVC-amorphous structure into a layered one. It is also a bi-axially oriented PVC that offers improved properties. Lastly, Modified PVC (PVC-M) is an alloy with modifying agents, toughness, and impact properties.

#### Others

The plastics included in the Others category are not classified into the former 6 types of common plastics: PET, HDPE, PVC, LDPE, PP and PS. This includes plastics that are resin-type, recyclable, non-recyclable, biodegradable or even mixed plastics. One of the most common types of Others category plastics is polycarbonates. Polycarbonates are thermoplastics made up of polymers with carbonate groups in their structure. Due to its properties similar to glass, it is translucent and can withstand high impacts while easily shaping at room temperature. It also has a natural UV light filter similar to glass. Due to that property, it is commonly used in glasses, eyewear or, protective gear. Although polycarbonates show excellent properties in replacing glasses, some studies show that polycarbonates release bisphenol A (BPA), which will result in a hazard for humans. Hence, this is the reason why polycarbonates are used less nowadays. Other polycarbonate products are bulletproof glass, car parts, CD and DVDs^21^.

Another common plastic in the Other category is Polylactic Acid (PLA). PLA is considered to be biodegradable plastic that is made up of cornstarch or sugar cane. It also has a low melting point and is insoluble in water. Due to its biodegradable property, it is commonly used in food packaging and 3D printing at home. Acrylonitrile Butadiene Styrene (ABS) is also another common type of plastic. ABS is made up of petroleum and has an opaque property. It is lightweight and strong. It also has good shock absorbency and high resistance against corrosive chemicals. Some common ABS products are Lego bricks, toys, computer keyboards and mouse^21^.

**Figure 1:**
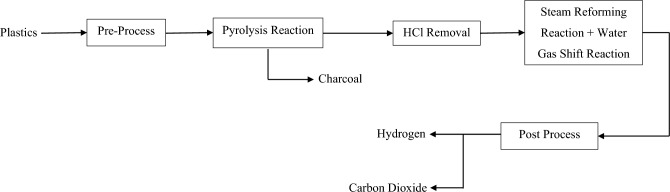
Block Flow Diagram of the process.

### Methods

#### Pre-process

Figure 1 shows the process of hydrogen production from plastic waste. Firstly, for the pre-processing, the plastic waste collected is crushed to reduce the plastic size to a minuscule, around 4 mm, to increase the heating rate and promote a fast pyrolysis reaction^22^. Nitrogen (N_2_) is then introduced to the plastics as a carrier gas to create an inert condition without oxygen. Nitrogen is obtained from air by separating nitrogen from dust and oxygen using an air filter and pressure swing adsorption (PSA) method.

#### Process

The pyrolysis reaction will occur in a conical sprout bed reactor (CSBR). In older days, a fixed bed reactor was used as the reactor. Still, it imposed problems such as uncompleted reactions due to the sticky nature of plastic and catalyst deactivation from the coke deposition. To prevent that problem, CSBR is proposed for pyrolysis reaction. CSBR provides cyclic and vigorous particle collision of a particle in different phases to prevent particle agglomeration in the bed matter, which prevents coke from depositing. It also has a high heat transfer rate that can enhance the pyrolysis reaction^23^. The particle’s cyclic movement produces a more uniform product^24^. The CSBR conditions are the temperature at 500 °C and pressure at 1 atm^22,23,25–27^. It also has a high heat transfer rate that can enhance the pyrolysis reaction^23^. The particle’s cyclic movement produces a more uniform product^24^. The CSBR conditions are the temperature at 500 °C and pressure at 1 atm^22,25,28,29^. After CSBR, a cyclone is introduced to remove solids and carbon formed during pyrolysis by utilizing the densities of gas and solid. Due to the presence of PVC plastic, the product streams of the pyrolysis reaction will consist of hydrochloric acid (HCl), a very strong acid. The presence of HCl will cause problems in the downstream equipment, such as separating hydrogen from other gases such as carbon dioxide and carbon monoxide^30^. This leads us to an immediate separation of HCl from the product stream of pyrolysis reactor to prevent problem stated above by introducing neutralization reaction by using sodium carbonate (Na_2_CO_3_). By removing hydrochloric acid, it also increases production yield of hydrogen as there are less contaminant. After separation of hydrochloric acid, a third reactor is introduced. A FBR is used for SRR and WGSR of the volatiles and gases from the product stream of the pyrolysis reactor. Instead of using fixed bed reactor in the older which imposed problem similar in the pyrolysis reactor which is the blocking of the bed due to coke deposition, FBR shows a better performance than fixed bed reactor due to the motion of the solid, which prevents the coke from deposit^25^. Steam is introduced here as raw material for SRR and WGSR. The condition used in the second FBR is the temperature at 700 °C and pressure at 1 atm^22,25,28,29^.

#### Post-process

A heat exchanger is introduced to reduce the temperature of the product stream to room temperature. A separator is then used to separate gases from liquids, such as water and heavy hydrocarbons, by utilizing the densities of gases and liquids. Here, the product stream consist mainly of hydrogen and carbon dioxide with few unreacted gases. To separate hydrogen from carbon dioxide, the PSA method is used. For this process, PSA is preferable to cryogenic distillation for certain reasons. Firstly, cryogenic distillation is used to separate all the syngas produced in the WGSR and SRR; PSA is mostly used to purify Hydrogen, which is the main focus of this project. Secondly, PSA is a much cheaper manufacturing cost than cryogenic distillation due to having much simpler technology for PSA than cryogenic distillation, which is more sophisticated. Due to this, very high pressure at 39.5 atm and temperature at 40 °C is utilized in PSA^31–34^. The disadvantages of cryogenic distillation include a high energy demand for regeneration, a high running cost, and a higher probability of process blockage. In the end stream of the process, hydrogen gas is obtained^35^.

### Reaction details

The details of the pyrolysis reaction are shown in Table 1, where each type of plastic (shown from raw materials) is undertaken pyrolysis reaction, which produces hydrocarbons, Hydrogen and Carbon. In addition, PVC, it produces Hydrochloric Acid (HCl). Table 2 show the details reaction for neutralization.

**Table 1:** Reaction details for pyrolysis reaction.

Parameter	Value
Naming code	R-101
Reactor type	Conical sprout bed reactor
Temperature	500 °C
Pressure	1 atm (101.325 kPa)
Reaction	PE^36^ $36{\text{C}}_{2}{{\text{H}}}_{4}\to {3}{\text{H}}_{2}+ \text{C} {\text{H}}_{4}\text{+}{\text{C}}_{2}{{\text{H}}}_{6}\text{+}{\text{C}}_{3}{{\text{H}}}_{6}\text{+}{\text{C}}_{3}{{\text{H}}}_{8}\text{+}{\text{C}}_{4}{{\text{H}}}_{10}\text{+}{\text{C}}_{8}{{\text{H}}}_{16}\text{+}{\text{C}}_{16}{{\text{H}}}_{32}\text{+}{\text{C}}_{28}{{\text{H}}}_{56}+ \text{7C}$ PP^36^ $29{\text{C}}_{3}{{\text{H}}}_{6}\to {19}{\text{H}}_{2}+ \text{C} {\text{H}}_{4}\text{+}{\text{C}}_{2}{{\text{H}}}_{6}\text{+}{\text{C}}_{2}{{\text{H}}}_{4}\text{+}{\text{C}}_{3}{{\text{H}}}_{8}\text{+}{\text{C}}_{4}{{\text{H}}}_{10}\text{+}{\text{C}}_{8}{{\text{H}}}_{16}\text{+}{\text{C}}_{16}{{\text{H}}}_{32}\text{+}{\text{C}}_{28}{{\text{H}}}_{56}+ \text{23C}$ PS^36^ $19{\text{C}}_{8}{{\text{H}}}_{8}\to {5}{\text{H}}_{2}+ \text{C} {\text{H}}_{4}\text{+}{\text{C}}_{2}{{\text{H}}}_{6}\text{+}{\text{C}}_{2}{{\text{H}}}_{4}\text{+}{\text{C}}_{3}{{\text{H}}}_{8}\text{+}{\text{C}}_{4}{{\text{H}}}_{10}\text{+}{\text{C}}_{8}{{\text{H}}}_{16}\text{+}{\text{C}}_{16}{{\text{H}}}_{32}\text{+}{\text{C}}_{28}{{\text{H}}}_{56}+ \text{85C}$ PET^37^ $3{\text{C}}_{10}{{\text{H}}}_{8}{{\text{O}}}_{4}\to {\text{C}}_{9}{{\text{H}}}_{8}{{\text{O}}}_{2}\text{+}{\text{C}}_{6}{{\text{H}}}_{6}\text{+}{\text{C}}_{6}{{\text{H}}}_{5}\text{COOH+CO+C}{\text{O}}_{2}\text{+}{\text{C}}_{2}{{\text{H}}}_{2}$ PVC^38^ ${\text{C}}_{2}{{\text{H}}}_{3}{\text{Cl}}\to {\text{C}}_{2}{{\text{H}}}_{2}+ \text{HCl}$

**Table 2:** Reaction details for neutralization.

Parameter	Value
Naming code	R-201
Reactor type	Fixed bed reactor
Temperature	500 °C
Pressure	1 atm (101.325 kPa)
Reaction	Neutralization^39^ $\text{HCl} + {\text{Na}}_{2}{\text{C}}{\text{O}}_{3}\to \text{NaCl} + {\text{H}}_{2}\text{O+C}{\text{O}}_{2}$

Due to the presence of Hydrochloric Acid, a neutralization process is needed to avoid any damage in the next reactor. Due to this, it then produces salt (${\text{NaCl}}$), including. ${\text{H}}_{2}{\text{O}}$ and ${\text{C}}{\text{O}}_{2}$, at 500 °C and 1 atm (101.325 kPa).

The hydrocarbons from the Pyrolysis reactor are treated, producing Carbon Monoxide and Hydrogen. Once this is produced, a water gas shift reaction occurs as shown in Table 3, producing ${\text{C}}{\text{O}}_{2}$ and ${\text{H}}_{2}$.

**Table 3:** Reaction details for steam reforming reaction and water gas shift reaction.

Parameter	Value
Naming code	R-301
Reactor type	Fluidized bed reactor
Temperature	700 °C
Pressure	1 atm (101.325 kPa)
Reaction	Steam reforming reaction^25^ CH_4_ ${\text{C}}{\text{H}}_{4}\text{+}{\text{H}}_{2}{\text{O}}\leftrightarrow \text{CO+3}{\text{H}}_{2}$ C_2_H_2_ ${\text{C}}_{2}{{\text{H}}}_{2}\text{+}{\text{2H}}_{2}{\text{O}}\leftrightarrow \text{2CO+3}{\text{H}}_{2}$ C_2_H_4_ ${\text{C}}_{2}{{\text{H}}}_{4}+ \text{2} {\text{H}}_{2}{\text{O}}\leftrightarrow \text{2CO+4}{\text{H}}_{2}$ C_2_H_6_ ${\text{C}}_{2}{{\text{H}}}_{6}+ \text{2} {\text{H}}_{2}{\text{O}}\leftrightarrow \text{2CO+5}{\text{H}}_{2}$ C_3_H_6_ ${\text{C}}_{3}{{\text{H}}}_{6}+ \text{3} {\text{H}}_{2}{\text{O}}\leftrightarrow \text{3CO+6}{\text{H}}_{2}$ C_3_H_8_ ${\text{C}}_{3}{{\text{H}}}_{8}+ \text{3} {\text{H}}_{2}{\text{O}}\leftrightarrow \text{3CO+7}{\text{H}}_{2}$ C_4_H_10_ ${\text{C}}_{4}{{\text{H}}}_{10}+ \text{3} {\text{H}}_{2}{\text{O}}\leftrightarrow \text{3CO+7}{\text{H}}_{2}$ C_6_H_6_ ${\text{C}}_{6}{{\text{H}}}_{6}+ \text{6} {\text{H}}_{2}{\text{O}}\leftrightarrow \text{6CO+9}{\text{H}}_{2}$ C_8_H_8_ ${\text{C}}_{8}{{\text{H}}}_{8}+ \text{6} {\text{H}}_{2}{\text{O}}\leftrightarrow \text{6CO+9}{\text{H}}_{2}$ C_8_H_16_ ${\text{C}}_{8}{{\text{H}}}_{16}+ \text{8} {\text{H}}_{2}{\text{O}}\leftrightarrow \text{8CO+16}{\text{H}}_{2}$ C_6_H_5_COOH ${\text{C}}_{6}{{\text{H}}}_{5}\text{COOH+12}{\text{H}}_{2}{\text{O}}\leftrightarrow \text{7CO+15}{\text{H}}_{2}$ C_9_H_8_O_2_ ${\text{C}}_{9}{{\text{H}}}_{8}{{\text{O}}}_{2}+ \text{16} {\text{H}}_{2}\leftrightarrow {\text{9C}}{\text{O}}_{2}+ \text{20} {\text{H}}_{2}$ C_10_H_8_O_4_ ${\text{C}}_{10}{{\text{H}}}_{8}{{\text{O}}}_{4}+ \text{16} {\text{H}}_{2}\leftrightarrow {\text{10C}}{\text{O}}_{2}+ \text{20} {\text{H}}_{2}$ C_16_H_32_ ${\text{C}}_{16}{{\text{H}}}_{32}+ \text{16} {\text{H}}_{2}{\text{O}}\leftrightarrow \text{16CO+32}{\text{H}}_{2}$ C_28_H_56_ ${\text{C}}_{28}{{\text{H}}}_{56}+ \text{28} {\text{H}}_{2}{\text{O}}\leftrightarrow \text{28CO+56}{\text{H}}_{2}$ Water gas shift reaction^25^ $\text{CO} + {\text{H}}_{2}{\text{O}}\leftrightarrow {\text{C}}{\text{O}}_{2}\text{+}{\text{H}}_{2}$

### Simulation diagram

**Figure 2:**
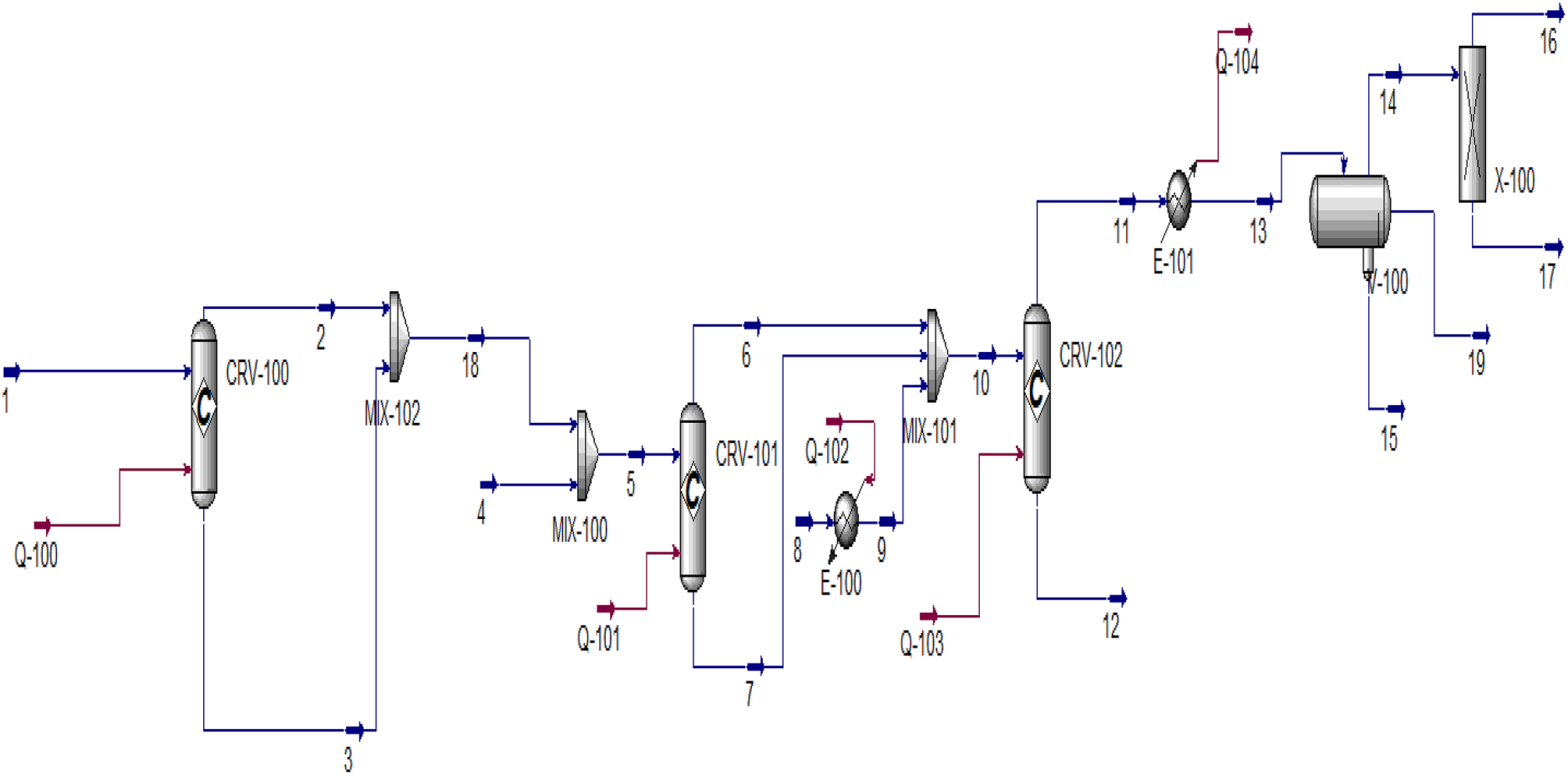
Aspen Hysys Simulation diagram for the prodcution of hydrogen from plastic waste.

Aspen Hysys (See Fig. 2) is being developed to simulate production of Hydrogen from plastic waste. This software is used because all major and minor equipments utilised for the production of hydrocarbons, Carbon dioxide and Hydrogen. Table 4 shows the major and minor equipment used in this study.

**Table 4:** Major and minor equipments desginated in Aspen Hysys.

Name	Function
CRV-100	Grinder and crusher
MIX-100	Mixer
E-100	Heat exchanger
CRV-101	Pyrolysis reactor
MIX-101	Mixer
CRV-102	Water gas shift reactor
E-101	Condenser
V-100	H_2_S and CO_2_ absorber

## Result and discussion

This section presents the outcomes manual for material and energy balance in each stream (both manual and simulation) and the percentage error between the results from manual calculation and simulation. Tables 5, 6 and 7 shows the manual calculation, simulation material balance and percentage difference between manual and simulation calculations for all the streams.

**Table 5:** Material balance manual calculations for all the streams.

Manual calculation	Stream number																
Mass flow of component (kg/h)	1	2	3	4	5	6	7	8	9	10	11	12	13	14	15	16	17
PE	4000.0		200.0														
PP	4000.0		200.0														
PS	4000.0		200.0														
PET	4000.0		200.0														
PVC	4000.0		200.0														
Hydrogen		126.6			126.6	126.6				126.6	4631.5		4631.5	4631.5	0.0	4631.0	0.5
Methane		141.0			141.0	141.0				141.0	7.0		7.0	7.0	0.0	0.0	7.0
Ethane		264.4			264.4	264.4				264.4	13.2		13.2	13.2	0.0	0.0	13.2
Ethene/ethylene		246.8			246.8	246.8				246.8	12.3		12.3	12.3	0.0	0.0	12.3
Propene		370.1			370.1	370.1				370.1	18.5		18.5	18	0.0	0.0	18.5
Propane		387.8			387.8	387.8				387.8	19.4		19.4	19.4	0.0	0.0	19.4
Butane		511.1			511.1	511.1				511.1	25.6		25.6	25.6	0.0	0.0	25.6
Octene		987.0			987.0	987.0				987.0	49.3		49.3	0.0	49.3		
C16H32		1974.0			1974.0	1974.0				1974.0	98.7		98.7	0.0	98.7		
C28H56		3463.4			3463.4	3463.4				3463.4	173.1		173.1	0.0	173.1		
Acetylene		1936.6			1936.6	1936.6				1936.6	96.8		96.8	96.8	0.0	0.0	96.8
Hydrochloric acid		2267.7			2267.7	113.4				113.4	113.4		113.4	113.4	0.0	0.0	113.4
Cinnamic acid		976.4			976.4	976.4				976.4	48.8		48.8	0.0	48.8		
Benzene		514.6			514.6	514.6				514.6	25.7		25.7	0.0	25.7		
Carbon dioxide		1161.1			1161.1	2442.0				2442.0	35,858.4		35,858.4	35,858.4	0.0	3.2	35,858.0
Water						524.0		28,719.2	28,719.2	29,243.2	1462.1		1462.1	0.0	1462.1		
Sodium chloride						3377.1				3377.1	3377.1		3377.1	0.0	3377.1		
Carbon monoxide											1036.0		1036.0	1036.0	0.00	0.0	1036.0
Benzoic acid			804.9														
Carbon			2936.5														
Sodium carbonate			–	3248.4	3248.4		162.42					162.4					
Total	20,000.0	15,328.7	4741.4	3248.4	18,577.1	18,356.5	162.4	28,719.2	28,719.2	47,075.7	47,067.3	162.4	47,067.3	41,832.3	5235.0	4634.3	37,200.8

**Table 6:** Simulation results from the Aspen Hysys for all the streams.

Simulation	Stream number																
Mass flow of component (kg/h)	1	2	3	4	5	6	7	8	9	10	11	12	13	14	15	16	17
PE	4000.0		200.0														
PP	4000.0		200.0														
PS	4000.0		200.0														
PVC	4000.0		200.0														
PET	4000.0		200.0														
Hydrogen		127.4			127.4	127.4				127.4	4657		4657.4	4657.4	0.0	4656.9	0.5
Methane		141.1			141.1	141.1				141.1	7.0		7.06	7	0.0	0.0	7.0
Ethane		264.5			264.5	264.5				264.5	13.2		13.2	13.2	0	0.0	13.2
Ethene/ethylene		246.8			246.8	246.8				246.8	12		12.3	12.3	0.0	0.0	12.3
Propane		387.9			387.9	387.9				387.9	19.4		19.4	19.4	0.0	0.0	19.4
Propene		370.2			370.2	370.2				370.2	18.5		18.5	18.5	0.0	0.0	18.5
Butane		511.3			511.3	511.3				511.3	25.6		25.6	25.6	0.0	0.0	25.6
Octene		987.1			987.1	987.1				987.1	49.36		49.4	48.7	0.49	-	48.7
C_16_H_32_		1974.4			1974.4	1974.4				1974.4	98.7		98.7	0.1	97.7	-	0.1
C_28_H_56_		3455.0			3455.0	3455.0				3455.0	172.7		172.7	172.7	0.0	-	172.7
Acetylene		1926.4			1926.4	1926.4				1926.4	96.3		96.3	96.3	0.0	0.0	96.3
Hydrochloric acid		2216.8			2216.8	110.8				110.8	110.8		110.8	110.8	0.0	0.0	110.8
Cinnamic acid		976.6			976.6	976.6				976.6	48.8		48.8	0.0	48.7	-	0.0
Benzene		514.8			514.8	514.8				514.8	25.7		25.7	25.7	0.0	–	25.7
Carbon dioxide		1160.4			1160.40	2431.40				2431.40	35,771.4		35,771.4	35,771.1	0.2	0.3	35,770.8
Water						520.2		28,743.3	28,743.3	29,263.6	1529.3		1529.32	786.9	742.4	–	786.9
Sodium chloride						3375.6				3375.6	3375.6		3375.6	0.0	3375.6	–	0.0
Carbon monoxide											1033.7		1033.7	1033.7	0.0	0.0	1033.7
Benzoic acid			804.1														
Carbon			2933.4														
Sodium carbonate				3222.1	3222.1		161.1			161.1		161.1					
Total	20,000.0	15,261.1	4738.9	3222.1	18,483.2	18,322.1	161.1	28,743.3	28,743.3	47,226.5	47,066.1	161.1	47,066.1	42,800.8	4265.3	4657.3	38,143.5

**Table 7:** Percentage error between Manual and simulation calculation for all the streams.

Percentage difference between manual calculation and simulation	Stream number																
	1	2	3	4	5	6	7	8	9	10	11	12	13	14	15	16	17
PE	0.0%		0.0%														
PP	0.0%		0.0%														
PS	0.0%		0.0%														
PVC	0.0%		0.0%														
PET	0.0%		0.0%														
Hydrogen		0.61%			0.61%	0.6%				0.6%	0.5%		0.5%	0.5%	100%	0.5%	0.5%
Methane		0.0%			0.0%	0.0%				0.0%	0.0%		0.0%	0.0%	100%	46.6%	0.0%
Ethane		0.0%			0.0%	0.0%				0.0%	0.0%		0.0%	0.0%	100%	6.7%	0.0%
Ethene/ethylene		0.0%			0.0%	0.0%				0.0%	0.0%		0.0%	0.0%	100%	33.3%	0.0%
Propane		4.6%			4.5%	4.5%				4.6%	4.5%		4.5%	4.5%	100%	0.04%	4.5%
Propene		4.5%			4.5%	4.5%				4.5%	4.5%		4.5%	4.5%	100%	27.5%	4.5%
Butane		0.0%			0.0%	0.0%				0.0%	0.0%		0.0%	0.0%	100%	93.8%	0.0%
Octene		0.0%			0.0%	0.0%				0.0%	0.0%		0.0%	100%	98.9%		
C_16_H_32_		0.0%			0.0%	0.0%				0.0%	0.0%		0.0%	100%	0.9%		
C_28_H_56_		0.2%			0.2%	0.2%				0.2%	0.2%		0.2%	100%	99.9%		
Acetylene		0.5%			0.5%	0.5%				0.5%	0.5%		0.5%	0.5%	100%	76.9%	0.5%
Hydrochloric acid		2.2%			2.2%	2.2%				2.2%	2.2%		2.2%	2.2%	100%	83.8%	2.2%
Cinnamic acid		0.0%			0.0%	0.0%				0.0%	0.0%		0.0%	100%	0.1%		
Benzene		0.0%			0.0%	0.0%				0.0%	0.0%		0.0%	100%	99.8%		
Carbon dioxide		0.0%			0.0%	0.4%				0.4%	0.2%		0.2%	0.2%	100%	88.8%	0.2%
Water						0.7%		0.0%	0.0%	0.0%	4.3%		4.3%	100%	49.2%		
Sodium chloride						0.0%				0.0%	0.0%		0.0%	100%	0.0%		
Carbon monoxide											0.2%		0.2%	0.2%	100%	6.9%	0.2%
Benzoic acid			0.015%														
Carbon			0.088%														
Sodium carbonate				0.809%	0.809%		0.809%										
Total	0.000%	0.441%	0.052%	0.809%	0.505%	0.187%	0.809%	0.084%	0.084%	0.319%	0.0%	0.8%	0.0%	2.2%	18.5%	0.4%	2.4%

Tables 8 show the energy calculation (manual and simulation) and percentage difference between both calculations. The streams have a satisfactory result of error percentage lower than 5% except for stream 15. This is due to the complex separation of gases and liquid and their interactions in the VKO drum that only Aspen HYSYS can simulate. In these calculations, due to unavailibility of data, 100% separation is assumed. Through simulation, it was observed that the separation of gases and liquid becomes more effective as the temperature decreases. However, considering cost and surrounding temperature, room temperature is chosen as the operating temperature.

**Table 8:** Heat flow values for both manual estimation and simulation results along with percentage error.

Stream	Heat flow (kJ/kmol)		Deviation
	Manual estimation	Aspen Hysys Simulation	
Q-100	12,197,660.60	12,938,950.00	5.7%
Q-101	− 53,610,017.50	− 53,130,740.00	0.9%
Q-102	97,007,089.60	96,992,660.00	0.0%
Q-103	94,675,443.00	97,957,510.00	3.4%
Q-104	102,542,556.30	94,435,290.00	8.6%

Energy calculations shows a satisfactory result for all the streams, which is lower than 10% of the difference. This shows that the energy balance calculations from Aspen HYSYS simulation can be used for better accuracy, rather than manual calculation.

From the plant design calculations with an input of 20,000 kg per hour of plastics, it is observed that 38,143.5 kg per hour of hydrogen with a purity of 99.99% can be obtained, which is equivalent to producing 340,000 tons of Hydrogen per year. Also, as a by-product, charcoal can be produced at a rate of 4,739 kg per hour, equivalent to around 42,000 tons per year. The cost estimation is calculated using standard equations given in Coulson and Richardson’s Chemical Engineering Design Volume 6. The total purchase cost of the equipment is given in Table 9, Inside battery limit (ISBL) plant cost is given in Table 10, fixed captial cost is given in Table 11, shown in Tables 9, 10, 11, 12, 13, 14, 15 and 16.

**Table 9:** Total purchase cost of the equipment (PCE).

Equipment no.	Cost (USD$ Dec 2022)	Cost (BND$ Dec 2022)
C-101	481,185.9	644,789.1
R-101	327,953.0	439,457.1
V-103	9574.1	12,829.3
R-201	267,401.9	358,318.6
E-301	100,915.5	135,226.8
R-301	1,048,345.9	1,404,783.5
E-302	100,915.5	135,226.8
V-301	29,215.1	39,148.3
V-302 &V-303	841,554.8	841,554.8
Total (PCE)	3,207,062.1	4,011,334.5

**Table 10:** Inside battery limit (ISBL) plant cost.

Item	Installation factor	Cost (BND$ Dec 2022)
PCE	1.0 × PCE	4,011,334.6
Equipment erection	0.3 × PCE	1,203,400.4
Piping	0.8 × PCE	3,209,067.7
Instrumentation and control	0.3 × PCE	1,203,400.4
Electrical	0.2 × PCE	802,266.9
Civil	0.3 × PCE	1,203,400.4
Structures and buildings	0.2 × PCE	802,266.9
Lagging and paint	0.1 × PCE	401,133.5
Total (ISBL)		12,836,270.6

**Table 11:** Fixed capital cost.

Item	Typical factor	Cost (BND$ Dec 2022)
ISBL	1 × ISBL	12,836,270.6
OSBL	0.3 × ISBL	3,850,881.2
D&E	0.3 × ISBL	3,850,881.2
X	0.1 × ISBL	1,283,627.0
Total (fixed capital investment)		21,821,660.1

**Table 12:** Total investment.

Item	Cost (BND$ Dec 2022)
Fixed capital investment	21,821,660.1
Working capital investment	2,567,254.1
Total investment	24,388,914.2

**Table 13:** Fixed cost of production.

Item	Factor	Cost (BND$ Dec 2022)
Operating labor (including overhead)	From estimation	630,000.0
Laboratory costs	0.2 × operating labor	126,000.0
Supervision	0.25 × operating labor	157,500.0
Plant overhead	0.5 × operating labor	315,000.0
Maintenance (including labour and materials)	0.1 × fixed capital	2,182,166.0
Capital charges	0.1 × fixed capital	2,182,166. 0
Taxes	0.01 × fixed capital	218,216.6
Insurances	0.01 × fixed capital	218,216.6
License fees and royalty	0.01 × fixed capital	218,216.6
Total		6,247,481.8

**Table 14:** Variable cost of production.

Item	Cost (BND$ Dec 2022)
Raw materials	
Waste plastics	215,986,560.0
Consumables	
Adsorbent	487,171.8
Catalyst	24.596.244.2
Utilities	
Water	58,958.7
Nitrogen	1,866,911.8
Shipping and packaging	
Shipping and packaging	Negligible
Total	242,995,846.7

## Cost analysis

To estimate the feasibility of the project, cost analysis is done using the factorial method to determine the purchase cost of equipment (PCE) and further estimate the Total Investment needed, Cash Cost of Production (CCOP) and Total Annual Revenue. At the end of this cost analysis, the return on investment and payback period are calculated to ensure the project can obtain profit at a reasonable rate. The cost analysis method is done with methods stated in Chemical Engineering Design—Principles, Practice and Economics of Plant and Process Design by Gavin Towler and Ray Sinnott and Coulson & Richardson’s Chemical Engineering Volume 6 Fourth Edition Chemical Engineering Design by R K Sinnott^40,41^.

## Total purchase cost of equipment (PCE)

This section will evaluate the total purchase cost of equipment (PCE). The value of each piece of equipment is estimated accordingly from Chemical Engineering Design—Principles, Practice and Economics of Plant and Process Design by Gavin Towler and Ray Sinnott. Furthermore, location factors are considered in the calculation, and CEPCI indices are carefully chosen to ensure the cost estimated is nearest to 2023. As CEPCI 2023 index is not yet released, CEPCI 2022 December is chosen^42^. The total purchase cost of the equipment is given in Table 9.

## Inside battery limit cost (ISBL)

The inside battery limit investment can be understood as the cost of the plant itself, including procuring and installing all equipment to make up the new plant. The ISBL cost includes equipment erection, piping, instrumentation and control, electrical, etc. The equation below can be used to calculate ISBL. The Inside battery limit (ISBL) plant cost is given in Table 10:

$$ISBL=PCE(1+{f}_{er}+{f}_{p}+{f}_{i}+{f}_{el}+{f}_{c}+{f}_{s}+{f}_{l})$$

where, $${f}_{er}=\text{Installation \, factor \, for \, Equipment \, Erection}$$, $${f}_{p}=\text{Installation \, factor \, for \, Piping}$$, $${f}_{i}=\text{Installation \, factor \, for \, Instrumentation \, and \, Control}$$, $${f}_{el}=\text{Installation \, factor \, for \, electrical \, work}$$, $${f}_{c}=\text{Installation \, factor \, for \, civil \, engineering \, work}$$, $${f}_{s}=I\text{nstallation \, factor \, for \, structures \, and \, buildings}$$, $${f}_{l}=\text{Installation \, factor \, for \, lagging, \, insulation \, and \, paint}$$.

## Fixed capital investment

The fixed capital investment can be defined as the sum of the cost for ISBL, Outside Battery Limit (OSBL), design and engineering (D&E), and contingency money (X). The listed items can be calculated as a factor of ISBL. OSBL is the cost of the additions needed to make to the site to accommodate building a new plant or increasing the capacity of the existing plant. D&E includes costs for the contractors and engineering services, such as detailed design for the plant. Contingency is the extra cost to ensure the project has more flexibility when changes are needed to ensure plant feasibility. OSBL, D&E, and X can be defined as factors of ISBL, as shown in the Table 11.

## Total investment

Total Investment can be defined as the sum of fixed capital investment and working capital investment, where the working capital investment is 20% of fixed capital investment. The total investment given in Table 12.

## Cash cost of production

Cash cost of production (CCOP) is the cost of production annually for this plant. It can be split into two groups: fixed cost of production (FCOP) and variable cost of production (VCOP). FCOP covers the exact cost regardless of the plant operation rate or output, while VCOP covers the cost directly tied to the production rate. The cash cost of production given in Table 15.

**Table 15:** Cash cost of production.

Item	Cost (BND$ Dec 2022)
FCOP	6,247,481.8
VCOP	242,995,846.7
CCOP	249,243,328.5

## Fixed cost of operation

FCOP cost includes most human-related works such as laboratory, supervision, plant overhead, maintenance, etc. All of the items can be defined as factors of operating labor. Fixed cost of production is given in Table 13.

## Variable cost of production

VCOP includes items directly affecting the production output rate, including raw materials, consumables, miscellaneous materials, utilities, shipping, and packaging. Typically, miscellaneous material costs are 10% of maintenance costs, and shipping and packaging costs are negligible. The variable cost of production is given in Table 14.

## Annual revenue

The main product of our plant is hydrogen, with char and heavy oil as the by-product. The selling price of hydrogen is BND$ 6.32/kg^43^. In addition, the selling price of char and heavy oil is BND$ 0.06/kg and BND$ 0.80/kg, respectively^44,45^. The summary of the total annual revenue is given in Table 16.

**Table 16:** Summary of the total annual revenue.

Component	Selling price (BND$/kg)	Production rate (kg/year)	Revenue (BND$/year)
Hydrogen	6.3	4314.1	240,732,279.8
Char	0.0	2717.75	1,428,445.6
Heavy oil	0.5	5763.3	25,278,084.5
Total			267,438,809.9

## Projected cash flow

**Figure 3:**
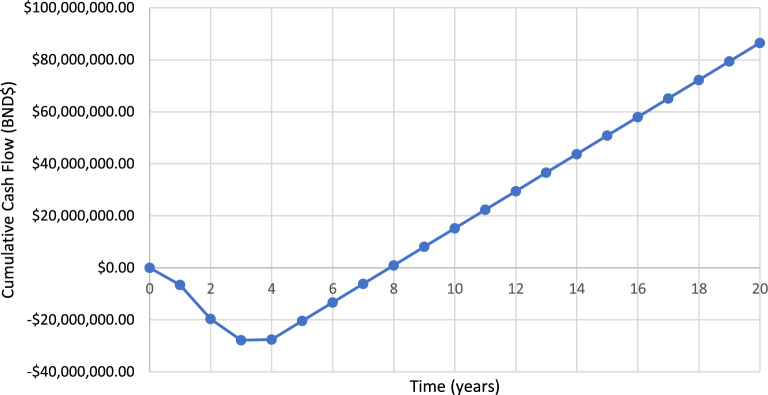
Cumulative cash flow against the time.

Using the cost economic data obtained above and the assumptions stated below, a cumulative cash flow is obtained as shown in Fig. 3.

Assumption considered:

- The plant takes 3 years to build.
- The tax rate is 30% of the annual profit, the interest rate is 6% of the total investment cost, and depreciation is 25%.
- Total fixed capital will be invested only in the first 3 years at a percentage of 30%, 60% and 10%, respectively, and total working will be invested only in the third year as the plant starts commissioning.
- Fixed cost of production will start to be invested in the third year, and variable cost of production will start at a rate of 30% in the third year, 60% in the fourth year and 100% in the fifth year and beyond.
- Plant commission starts at a rate of 30% in the third year, 60% in the fourth year, and 100% in the fifth year and beyond, corresponding to the variable cost of production.
- The annual revenue and cash cost of production cost will remain constant starting the fifth year.
- Cash flow analysis is done for the first 20 years only.

## Profitability analysis

### Payback time

Payback time is the year taken to recover back the total investment cost. It can be calculated from the cumulative cash flow diagram using the interpolation method to estimate the payback time. The cumulative cash flow is zero at the payback time when the money starts to break even. From the graph, the following information can be obtained, for year 8 & 9, the cumulative cash flow (BND $) are − 6,148,236.45 and 979,150.24 respectively.

Hence, the payback time for this project is around 7.86 years.

## Return on investment

Return on investment (ROI) is an analysis to measure the efficiency of an investment in a project. It defines the economic benefits that can be obtained concerning its cost. The following equation is used to calculate the ROI of a project:

$$ROI=\frac{cumulative \, net \, cash \, flow \, at \, end \, of \, project}{plant \, life\times total \, investment}$$

$$ROI=\frac{86507790.42}{20 \, years\times 24388914.24}$$

$$ROI=17.74\%$$

Hence, the ROI for this project at the end of 20 years is 17.74%.

## Net present value

Net present value (NPV) is a more useful economic analysis compared to ROI and payback time as it considers the time value of money and accounts for annual variation in expense and revenues to determine the value of the investment. The following equation can be used to find out the NPV of a project:

$$NPV=\sum_{n=1}^{n=t} \frac{{CF}_{n}}{{(1+i)}^{n}}$$

where, $${CF}_{n}=\text{cash flow in a year n, n is project life in years}$$, $$\text{t is the interest rate=6}\%$$.

$$NPV=\sum_{n=20}^{n} \frac{{CF}_{20}}{{(1+0.06)}^{20}}$$

$$NPV=\frac{-6546498.03}{{(1+0.06)}^{1}}+\frac{-13092996.06}{{(1+0.06)}^{2}}+\frac{-8174259.38}{{(1+0.06)}^{3}}+\dots +\frac{7127386.68}{{(1+0.06)}^{20}}$$

$$NPV=BND\$ \mathrm{35,491,210.84}$$

If the plant is continued to work for 20 years, the NPV of this plant is BND$ 35,491,210.84. This positive value of NPV means that this project is worth the investment even through its take 20 years of the operation period. However, every process plant is designed to work atleast for 50 years with minor design changes, as old equipments are replaced with latest sofisticated equipments. 

## Sensitive analysis

To further understand the cost analysis of the project, sensitive analysis is done by modifying variables that affect the cost and revenue of the plant. This ensures that when the economy is not favorable towards hydrogen production, this project can still gain profit and further understand how the economy affects the plant's revenue plant. In this sensitive analysis, the following scenarios are conducted, as shown in Tables 17 and 18.

**Table 17:** Scenarios designed for the best and worst case based on the base case.

Variable	Best case (% of base case)	Worst case (% of base case)
Sale price	101%	99%
ISBL cost	95%	105%
OSBL cost	95%	105%
Production rate	101%	99%

**Table 18:** Analysis of scenarios (worst, base, and best cases).

	Worst case	Base case	Best case
ISBL cost (BND $)	13,478,084.1	12,836,270.6	12,194,457.1
OSBL cost (BND $)	4,245,596.5	3,850,881.2	3,475,420.2
Fixed capital investment (BND $)	23,114,914.3	21,821,660.1	20,547,660.2
Total amount of investment (BND $)	25,810,531.2	24,388,914.2	22,986,551.6
FCOP (BND $)	6,544,930.3	6,247,481.8	5,954,461.8
VCOP (BND $)	242,995,846.7	242,995,846.7	242,995,846.7
CCOP (BND $)	249,540,777.0	249,243,328.5	248,950,308.5
Total annual revenue (BND $)	264,771,107.7	267,438,809.9	270,119,883.9
Payback time (year)	10.8	7.8	6.4
ROI (%)	8.2%	17.7%	28.3%
NPV (BND $)	10,008,608.4	35,491,210.8	62,246,772.7

## Conclusion

The negative repercussions of climate change are already being felt. Toxic compounds are emitted during the combustion of plastics, open burning, and incineration, causing harm to the natural atmosphere, particularly plants, and people's health. Proper policy formulation regarding chemical exposure produced by plastic must be implemented. A long-term step toward a healthier and cleaner environment is urgently needed. This would assist the general public in becoming aware of the seriousness of the situation and choosing technology that poses fewer risks to human health in developing countries. As a result, the scientific community must consider the cumulative environmental contamination that may affect human health. Instead of incineration and combustion, pyrolysis is an alternate approach that has been shown to create less hazardous compounds with varying levels of potentially beneficial by-products when conditions are favorable. Recycling reduces resource stress while also using byproducts, boosting sustainability. Implementing recycling programs and doing research will make a significant difference. Pyrolysis in a CSBR is a viable option for producing Hydrogen gas from plastic wastes. The generated simulated model for converting plastic wastes to hydrogen was conducted with the Aspen Hysys simulator’s assistance. The results were in satisfactory and a pilot case study will give more accurate results. This project feasibility study indicates that the different kinds of plastic waste procured (total input of 20,000 kg per hour of plastics) from Brunei Darussalam, Malaysia and Indonesia, can produce about 340,000 tons of Hydrogen per year. Furthermore, the price of hydrogen has an annual profit that ranges from $ 271,158,100 to $ 358,480,200. Moreover, producing hydrogen is an excellent alternative to eliminating pollution due to plastic waste consumption and as a replacement for oil and gas, the main energy resources in Brunei Darussalam. SDG 13 is climate action. Hence, with the Brunei Vision 2035 rising, it aims to ensure carbon control and focus more on renewable energy rather than oil and gas as the main energy resource.

## Data Availability

The datasets used and analyzed during the current study are available from the corresponding author upon reasonable request.
